# Predicting Kidney Graft Survival Using Machine Learning Methods: Prediction Model Development and Feature Significance Analysis Study

**DOI:** 10.2196/26843

**Published:** 2021-08-27

**Authors:** Syed Asil Ali Naqvi, Karthik Tennankore, Amanda Vinson, Patrice C Roy, Syed Sibte Raza Abidi

**Affiliations:** 1 Department of Computer Science Dalhousie University Halifax, NS Canada; 2 Division of Nephrology Dalhousie University Halifax, NS Canada

**Keywords:** kidney transplantation, machine learning, predictive modeling, survival prediction, dimensionality reduction, feature sensitivity analysis

## Abstract

**Background:**

Kidney transplantation is the optimal treatment for patients with end-stage renal disease. Short- and long-term kidney graft survival is influenced by a number of donor and recipient factors. Predicting the success of kidney transplantation is important for optimizing kidney allocation.

**Objective:**

The aim of this study was to predict the risk of kidney graft failure across three temporal cohorts (within 1 year, within 5 years, and after 5 years following a transplant) based on donor and recipient characteristics. We analyzed a large data set comprising over 50,000 kidney transplants covering an approximate 20-year period.

**Methods:**

We applied machine learning–based classification algorithms to develop prediction models for the risk of graft failure for three different temporal cohorts. Deep learning–based autoencoders were applied for data dimensionality reduction, which improved the prediction performance. The influence of features on graft survival for each cohort was studied by investigating a new nonoverlapping patient stratification approach.

**Results:**

Our models predicted graft survival with area under the curve scores of 82% within 1 year, 69% within 5 years, and 81% within 17 years. The feature importance analysis elucidated the varying influence of clinical features on graft survival across the three different temporal cohorts.

**Conclusions:**

In this study, we applied machine learning to develop risk prediction models for graft failure that demonstrated a high level of prediction performance. Acknowledging that these models performed better than those reported in the literature for existing risk prediction tools, future studies will focus on how best to incorporate these prediction models into clinical care algorithms to optimize the long-term health of kidney recipients.

## Introduction

### Background

Kidneys are vital for the health of an individual, as they filter waste products from the blood and produce hormones and urine [1]. Patients are considered to have end-stage renal disease when their kidney function falls below a specific threshold [2]. A lack of timely measures to prevent kidney failure results in premature death [3,4].

Kidney transplantation [5,6] and dialysis are the two main treatments for kidney failure [7]. Kidney transplantation offers a survival advantage compared with other forms of kidney replacement therapy; however, the rate of graft loss following transplant is still undesirably high [8]. Kidneys are a limited resource, and optimizing the match between donors and recipients is crucial for improving outcomes after transplantation. Kidney transplant allocation is, in part, based on a number of donor-recipient–related factors that influence graft survival. Various clinical studies have been conducted on the influence of these factors on graft survival; however, given the complex interactions between these factors, there remains much to be learned in this area. Existing risk prediction models only have a limited ability to predict outcomes for kidney transplant recipients with receiver operating characteristic scores of 0.6-0.7 [9-11].

Prediction modeling using machine learning (ML) algorithms has gained attention in recent years [12] for predicting the success of clinical or surgical procedures (such as kidney transplant). ML algorithms autonomously learn the underlying associations between preprocedure clinical features and postprocedural outcomes to predict the outcome of the procedure for a given clinical case. In kidney transplant, ML-based prediction models, based on donor-recipient information, autonomously learn the underlying relationships between donor and recipient factors to predict transplant outcomes. Multiple studies have been conducted using ML methods to predict the kidney graft outcome [13-16]; however, the standard approach in nearly all the reviewed studies has been to select one or more arbitrary period starting from the date of transplant and applying classification algorithms for prediction. There is a clear need for further exploration of data stratification approaches and other ML methods with respect to feature engineering and prediction modeling.

### Objectives

In this study, the intent is to investigate kidney transplant allograft survival, that is, estimating the time-to-event and the evolving influence of clinical features leading to an event—within three temporal cohorts of 1 year, >1-5 years, and >5 years of a kidney transplant. We predicted the outcome of graft failure after kidney transplant based on the analysis of donor and recipient features. We applied ML methods to (1) predict the graft status over different temporal periods and (2) analyze the changing effect of donor-recipient–related predictors across different periods. To develop the prediction models, we analyzed a large data set of over 50,000 transplants covering approximately a 20-year period of kidney transplants in the United States. To generate the clinically meaningful temporal cohorts, we experimented with two patient stratification approaches: (1) a novel *nonoverlapping* patient stratification approach, whereby a patient’s graft failure was recorded only in the temporal cohort when it actually happened, that is, a graft failure event in the preceding cohort was not included and (2) the traditional *overlapping* patient stratification approach that provides an accumulative count of graft outcomes until a specific time point. To develop the prediction models, we investigated multiple ML algorithms using both patient stratification approaches. Nonoverlapping temporal cohorts were considered to investigate the influence of clinical features on predicting graft survival over time, as the temporal partitioning of the data allowed for the establishment of feature influence across distinct temporal windows. We applied the feature importance method based on the mean decrease in impurity (Gini).

The contributions of this research are as follows: (1) ML-based prediction models that are trained on a large data set, offering improved prediction performance compared with previous studies (previous graft prediction studies are based on a smaller number of transplants over a shorter period); (2) data dimensionality reduction based on a deep learning framework to handle the high-dimensional and complex kidney transplant data set; (3) a novel nonoverlapping patient stratification approach to provide fine-grained feature importance within a specific period while avoiding bias from preceding cohorts; (4) explaining the influence of the different clinical features, during different periods, toward the prediction performance of ML prediction models. This finding allows the selection of the most important features to predict graft outcomes within a specific temporal window; and (5) a comparison between the two stratification approaches with respect to the performance of the prediction models. The future practical outcome of this study is the provision of a data-driven decision support tool to assist nephrologists in the kidney allocation process by identifying the best donor and recipient pair that will lead to the highest likelihood of graft survival for a given recipient.

### Prior Work

Patients can receive a kidney from either deceased donors or living donors. The donor-recipient matching process becomes relatively more complex with deceased donors because of the need to account for additional clinical factors (ie, prolonged cold ischemia time, prolonged wait times, and generally lower quality organs) [17]. Given the fact that kidney organs are a limited resource, it is important to have an efficient and effective donor-recipient matching process to ensure long-term graft survival [18].

Data-driven methods are now being used for organ matching; these methods are used to establish clinical compatibility beyond the blood group and tissue type. Conventional data-driven prediction methods use statistical techniques such as Cox proportional hazard models and Kaplan-Meier estimates to perform time-to-event analyses [19]. Significant research has been conducted with Cox-based models in the survival analysis of different organ transplants; however, these methods eventually lose predictive accuracy as the feature space increases [13,14].

ML-based data analysis to develop prediction models for predicting outcomes is usually performed using classification methods, whereas regression methods are used for time-to-event analysis. There are two prominent approaches to predict kidney allograft outcomes using ML-based classification methods. The first approach is to predict graft survival over time by dividing a longitudinal data set into different time cohorts based on the occurrence of a given adverse event or the last follow-up date from the date of transplant. Each time cohort has a binary target variable, that is, success or failure of the graft, which is used to train the classification model to predict graft survival [15,16]. The second approach is to predict the risk acuity associated with a graft within a period. Topuz et al [15] used this approach and divided the data set into three graft failure risk groups (high, medium, and low) across three different periods to predict the risk of graft failure within a specific time using classification methods. Li et al [20] used Bayes net to classify graft risk levels and predict graft survival.

Due to the high dimensionality of existing data sets for organ transplantation, feature selection is applied to filter out redundant features. A stacked autoencoder, which is an unsupervised neural network, is an efficient dimensionality reduction technique with promising performance for deep representation of medical data [21] that reconstructs its own inputs by first encoding them to a smaller size and then decoding back to the original inputs. A comparative study by Sadati et al [22] highlighted the efficacy of different types of autoencoders for data sets based on electronic health records.

Right-censored data are a common problem for survival analysis, as it represents cases for which the adverse event is not available or recorded because of either the subject having been lost to follow-up or not experiencing the event during the study period. Multiple approaches have been adopted in previous studies to address this problem. The study on kidney transplants by Topuz et al [15] discarded all the right-censored data before 7 years from the time of transplant and included the remaining transplants that took place after that time point in the low-risk group. In a study predicting heart transplant outcomes, the data set was divided into three different time cohorts (1, 5, and 9 years) to predict the status of the graft. Patients who did not have graft failure during a particular time cohort were censored, and all the patients beyond that time cohort were considered to have successful transplants [23].

The influence of clinical features (or clinical predictors) on graft survival tends to vary over time [16], as shown using a heat map [24]. Dag et al [23] analyzed the changing significance of features for three overlapping time cohorts (1, 5, and 9 years). They deduced that certain types of features perform well in the long term compared with the short and medium terms. For instance, socioeconomic factors were more influential in their 9-year time cohort as they covered major variations in the data. It is important to note here that feature significance over time can only be substantiated if the analysis is performed with nonoverlapping cohorts to avoid any bias introduced by the cumulative effect of data before the analysis period.

In previous studies [16,20-22,25], predicting graft failure has been pursued by taking an overlapping patient stratification approach, which means that each subsequent time cohort includes data from the previous cohort. This introduces a cumulative effect that is useful for predicting graft failure across a staggered time period. However, the overlapping patient stratification approach is ineffective in determining the influence of clinical features during a specific time period. Hence, the nonoverlapped cohort approach offers a novel mechanism to investigate the influence of clinical features within specific time windows. To the best of our knowledge, nonoverlapping cohorts have not been studied in the literature to develop prediction models or analyze the temporal influence of clinical features on kidney transplant outcomes.

This study is organized into five major sections: *Methods* presents the study’s methodology; *Results* presents the results of the prediction; and *Discussion* discusses the significance of clinical features toward graft status prediction across different time cohorts and offers a conclusion and future research directions.

## Methods

### Overview

**Figure 1:**
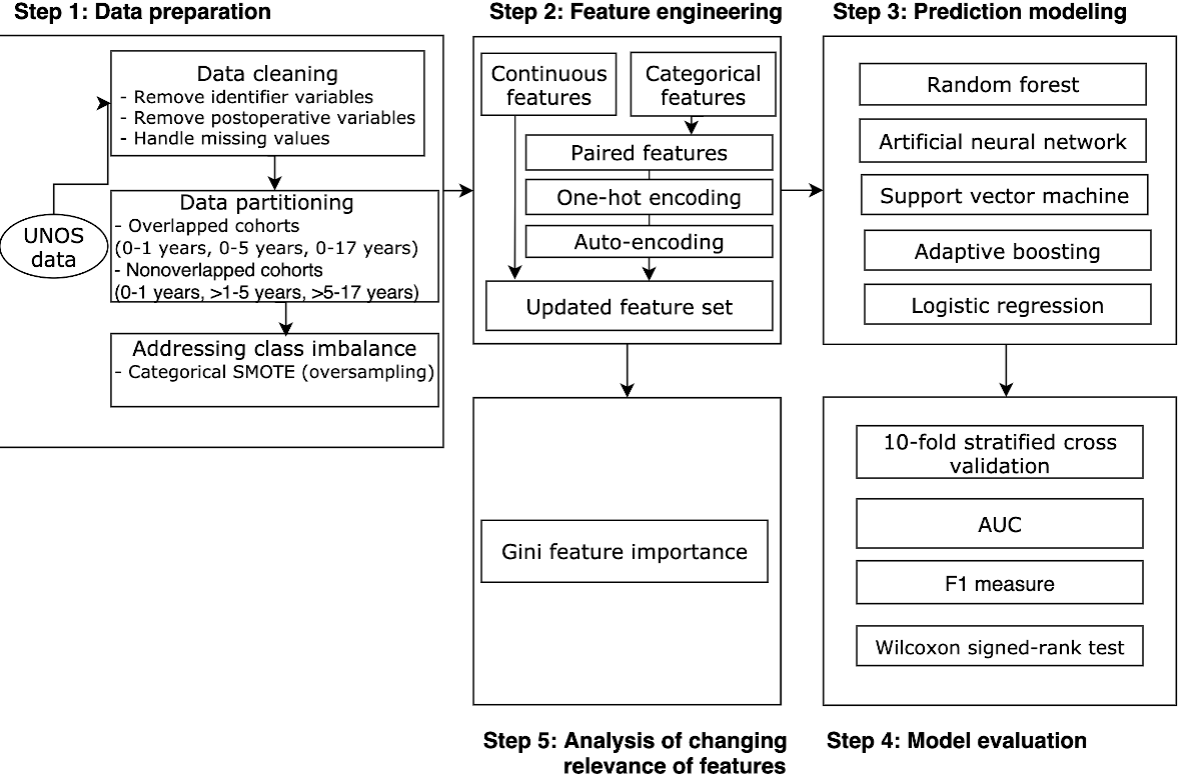
Overview of our data analytics methodology. AUC: area under the curve; SMOTE: synthetic minority oversampling technique; UNOS: united network of organ sharing.

To predict graft survival over time and to analyze the influence of clinical features on graft survival, our data analytics methodology (Figure 1) comprised data preparation, feature engineering, prediction modeling, model evaluation, and analysis of changing relevance of features.

### Data Description

This study used data from the Scientific Registry of Transplant Recipients (SRTR). The SRTR data system includes data on all donors, wait-listed candidates, and transplant recipients in the United States, submitted by the members of the Organ Procurement and Transplantation Network. The Health Resources and Services Administration and the US Department of Health and Human Services provided an overview of the activities of the Organ Procurement and Transplantation Network and SRTR contractors.

The data set provided pretransplant clinical features and outcomes of 277,316 kidney transplants between 2000 and 2017. Survival was reported in terms of graft outcome and patient status. For the purposes of this study, graft failure was defined as (1) graft loss or (2) death with a functioning graft.

We analyzed the data and used only complete cases (ie, no missing feature values), which comprised a total of 52,827 kidney transplants. Table 1 provides a list of the included clinical features and their descriptions used in our experiments.

**Table 1:** List of clinical features used to train the prediction models.

Feature description	Data type	Abbreviation
Peak panel reactive antibody	Continuous	PKPRA
Type of transplant	Categorical	REC_TX_PROCEDURE
Any previous kidney transplant	Categorical	PREVKI
Donor age	Continuous	DAGE
Donor height	Continuous	DHT100
Recipient height	Continuous	RHT2100
Donor weight	Continuous	DWT
Recipient weight	Continuous	RWT2
Donor creatinine level	Continuous	DONCREAT
Expanded criteria donor	Categorical	ECD
Donation after cardiac death	Categorical	DCD
Donor hypertension	Categorical	DHTN2
Recipient hypertension	Categorical	RHTN
Recipient BMI	Continuous	RBMI2
Donor BMI	Continuous	DBMI
Cold ischemia time	Continuous	CIT
Recipient age	Continuous	RAGETX
Number of HLA antigen mismatches (paired)	Categorical	HLAMM
Functional status of the recipient	Categorical	FUNCTSTAT
Donor-recipient sex (paired)	Categorical	DRSEX
Donor-recipient race (paired)	Categorical	DRRACE
Donor-recipient age (paired)	Categorical	DRAGE
Recipient cardiovascular disease	Categorical	RCVD
Donor hepatitis C virus	Categorical	DHCV
Recipient peripheral vascular disease	Categorical	RPVD
Donor race	Categorical	DRACESIMP
Recipient race	Categorical	RRACESIMP
Recipient malignancy	Categorical	RMALIG
Years on dialysis pretransplant	Continuous	VINTAGE
Donor diabetes	Categorical	DDM
Preemptive transplant	Categorical	PREEMPTIVE
Recipient diabetes	Categorical	RDM2
Recipient coronary artery disease	Categorical	RCAD
Simplified ESRD^a^ diagnosis	Categorical	ESRDDXSIMP
Donor-recipient CMV^b^ (paired)	Categorical	DRCMV
Donor-recipient height difference	Categorical	AHD1
Donor-recipient weight difference	Categorical	DRWT

^a^ESRD: end-stage renal disease.

^b^CMV: cytomegalovirus.

### Data Preparation

Data preparation for learning the ML-based prediction models consisted of data cleaning, partitioning the data set into temporal cohorts, and addressing class imbalances.

#### Data Cleaning

Data cleaning involved removing (1) all patient identifying features (such as transplant ID, donor ID, and patient ID) [23], (2) post- and intraoperative features (such as delayed graft function and warm ischemia time) as we focused on pretransplant features to predict outcomes [23], (3) living donors [15,26], (4) recipients below the age of 18 years [14,27,28], and (5) all sequential and en bloc transplants, both of which are atypical procedures that would not broadly apply to most deceased donor situations. These exclusion criteria were suggested by domain experts and also noted in prior studies [9,28].

#### Data Partitioning Into Temporal Cohorts

**Figure 2:**
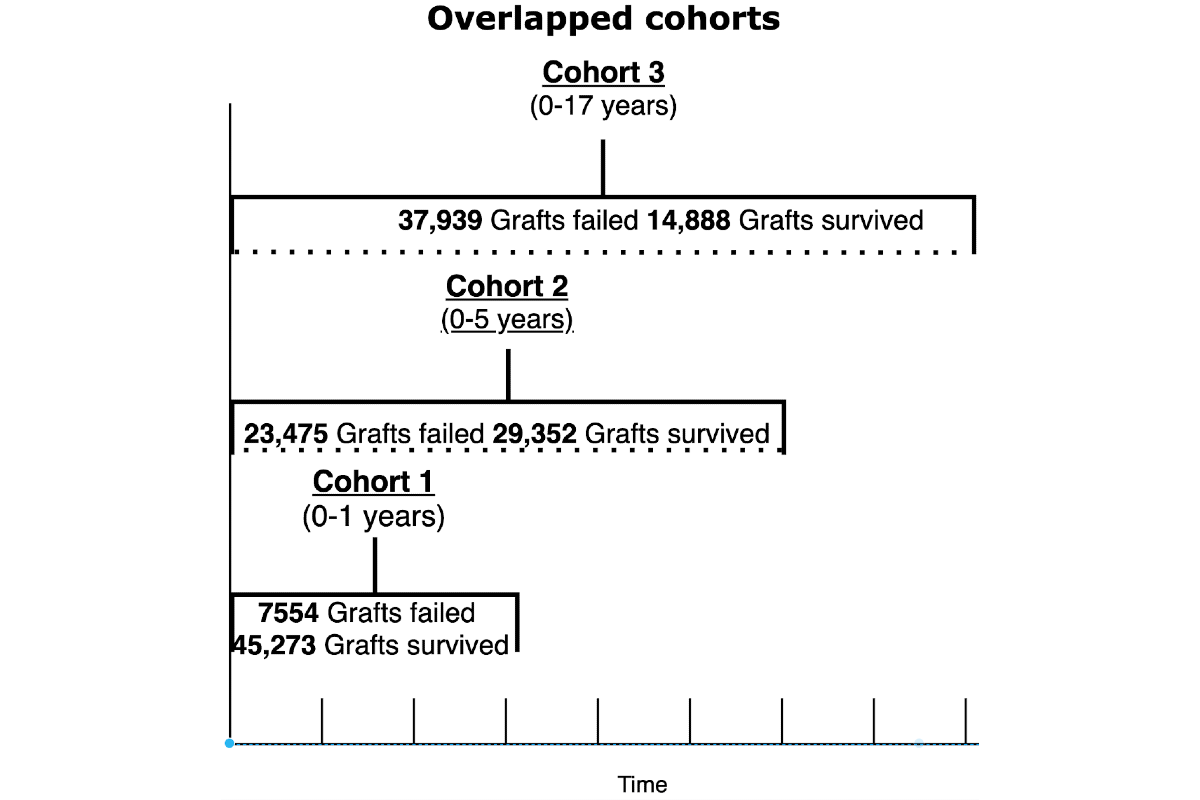
Derivation of the overlapped cohorts.

**Figure 3:**
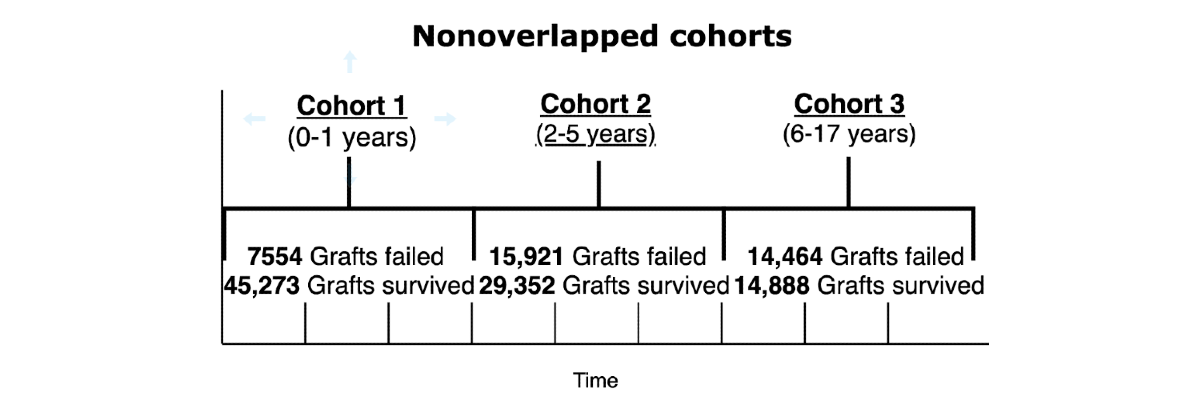
Derivation of the nonoverlapped cohorts.

Given the longitudinal data set, we generated two distinct data sets using traditional *overlapping* and our novel *nonoverlapping* patient stratification approaches to partition the data set into three temporal cohorts representing graft status at 1 year, >1-5 years, and 5-17 years (Figures 2 and 3).

The overlapping patient stratification approach (used in previous graft status prediction studies) provides a cumulative analysis of graft outcomes up to a specific time point. In our study, the overlapping data stratification resulted in the following three cohorts: cohort 1, spanning from year_0_ to year_1_, which reported graft outcomes (ie, graft failure or survival) during this period; cohort 2, which reported graft status from year_0_ to year_5_ and overlapped with cohort 1 such that it included the patients in cohort 1*;* and cohort 3*,* which reported graft status from year_0_ to year_10+_, thus overlapping with both earlier cohorts. As per the overlapping approach, a graft failure in the preceding cohort was also counted in the proceeding cohort.

Our nonoverlapping patient stratification approach yielded three cohorts: cohort 1*,* spanning year_0_ to year_1,_ reporting graft outcomes in this period; cohort 2, spanning year_1_ to year_5_ and reporting graft outcomes only in this period, resulting in the exclusion of graft failures reported in cohort 1 and only reporting the graft outcomes of patients who survived cohort 1*;* and cohort 3, spanning year_5_ to year_10+_, reporting the graft outcomes of patients who survived cohort 2*.* In a nonoverlapping cohort approach, there was no looking back beyond the cohort’s starting point, as such a graft failure in the preceding cohort was not counted in the proceeding cohort.

When partitioning the data into cohorts, we accounted for the presence of censored data, that is, the lack of information about the occurrence of an adverse event for a surviving patient. There is no concrete method to determine survivors when confronted with censored data. We initially assumed that those patients who did not fail in a certain cohort could be presumed as survivors. However, this assumption led to two problems: (1) it included censored patients who might have experienced graft failure during the study, and (2) it led to a severe class imbalance between the graft failure and surviving patients. To overcome these problems, we took a two-phase heuristic approach to remove the censored observations to identify *survivors* in each cohort. First, we removed all the censored observations from the cohort being analyzed and labeled all the remaining instances as survived. For instance, the censored data that were removed for cohort 2 were all instances with a missing outcome by the end of cohort 2. The remaining instances were considered to have survived. The first phase of our approach reduced the number of censored observations, but the surviving observations were still relatively high compared with the graft failure cases in each cohort. In the second phase, we applied the approach of Topuz et al [15] to further refine our surviving class by removing all the instances that were deemed as surviving for less than 8 years from the date of transplant. This two-phase approach to account for censored data is summarized in Equation 1 below, where we estimated the number of survivors in each cohort.

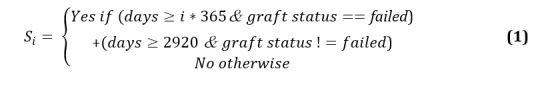



The first part of this equation illustrates the first phase of the proposed approach. The *i* in the equation is the ending year of our defined cohorts, that is, 1, 5, and 17. The equation first calculates the total number of graft failures that occurred after the end of the cohort. This fraction of instances was considered as confirmed survivors for the cohort under analysis. The second part of the equation deals with the second phase of the approach. It attempts to identify the potential survivors from the censored data by removing all the observations that did not have a graft failure within 8 years (2920 days) following a transplant. Table 2 shows the patient distribution across the three time cohorts.

**Table 2:** Number of failed and survived transplants in overlapped and nonoverlapped cohorts.

Cohort	Overlapping				Nonoverlapping		
	Count, n	Failed, n (%)	Survived, n (%)	Count, n		Failed, n (%)	Survived, n (%)
1	52,827	7554 (14.3)	45,273 (85.7)	52,827		7554 (14.3)	45,273 (85.7)
2	52,827	23,475 (44.44)	29,352 (55.56)	45,273		15,921 (35.17)	29,352 (64.83)
3	52,827	37,939 (71.82)	14,888 (28.18)	29,352		14,464 (49.28)	14,888 (50.72)

### Addressing Class Imbalance

Our data set had two outcomes: the presence or absence of graft failure. There was a significant class imbalance whereby the *graft failure* had a significantly lower number of instances compared with *graft survival.* Techniques such as Synthetic Minority Oversampling Technique (SMOTE) and random undersampling have been widely used in the literature [18,29] for class imbalance. We applied SMOTE for Nominal and Continuous features [30], which is a variant of SMOTE specifically developed to handle a mix of categorical and numerical data, on all cohorts to achieve a reasonable class balance. To balance the minority class (ie, mostly *graft failure*), we would need to generate 600% additional synthetic samples (at least for cohort 1), which would have led to overfitting. Therefore, we doubled our minority class to achieve a workable class balance to prevent the overfitting of the classification models. As cohort 2 of overlapped stratification and cohort 3 of nonoverlapped stratification had a class balance, they were not considered for oversampling.

### Feature Engineering

This step involved both the removal and construction of features with the intent to reduce the dimensionality of the feature space.

#### Paired Features

A set of paired features was constructed by combining the related features. Typically, graft predictions use discrete individual donor and recipient features. We examined the underlying correlation between the donor and recipient features and paired the highly related features to generate new *paired features*. The following four donor and recipient features were generated: sex, age, CMV (cytomegalovirus), and race. In addition, three types of HLA Antigen Mismatch features—ie, HLA Antigen Mismatch at the A Locus, HLA Antigen Mismatch at the B Locus, and HLA Antigen Mismatch at the DR Locus were also combined into a single HLA Antigen Mismatch feature.

#### One-Hot Encoding

We transformed the categorical features into multiple dummy features to make them compatible with the stacked autoencoders, which cannot process categorical features. In addition, it was also a necessary operation because of the functional constraint of the scikit-learn library [31].

#### Stacked Autoencoders

Finally, we used 86 transformed categorical features as inputs to the stacked autoencoders for feature reduction to subsequently train the ML prediction models. Continuous features were also initially considered as a part of the input vector to stacked autoencoders (Table S1 in Multimedia Appendix 1), but preliminary model training returned better results with pristine continuous features; hence, no modification was performed for them while training the prediction models. It should be noted that the resultant features from the stacked autoencoders were only used for training the prediction models and not for the analysis of the changing relevance of features over time.

After testing with different configuration settings provided in the Keras framework [32], the stacked autoencoders were set up as a 13-layer architecture consisting of 12 dense layers and one dropout layer set at the very beginning of the network with a dropout rate of 0.05. The sigmoid activation function was used throughout the dense layers, with Adam as the optimizer and binary cross entropy as the loss function with 500 epochs and 700-900 batch sizes. The middle layer of the autoencoder was finally trained with 12 neurons and 30 neurons for cohort 1 and the remaining cohorts, respectively. The feature space was reduced by more than 50 dummy features.

### Learning Classification-Based Graft Survival Prediction Models

#### Overview

Prediction was pursued as a binary classification problem, where the prediction output represents the graft outcome for a given patient in terms of the class label, graft failure or survived. We investigated four different ML-based classification models for each time cohort (ie, cohorts 1-3). Given that logistic regression (LR) has been widely used in prior studies to develop graft prediction models [29,33], we trained an LR model as a baseline to compare the predictive performance of our ML prediction models.

All classification models were trained using a 10-fold stratified cross-validation training approach. The stratification ensured that outcome class ratio in each fold is maintained to avoid any sampling bias that may affect the classification results. We mainly used the scikit-learn library [31] to train the below-mentioned classification models with the parameter settings listed in Table 3. Hyperparameters were optimized using a random search. The different parameters that were tested during the random search are provided in Table S2 in Multimedia Appendix 1.

**Table 3:** Algorithmic settings for the classifiers.

Method, Hyperparameter		Values
**RF^a^**		
	Number of estimators	200
	Class weight	Balanced
	Criterion	Gini
	Maximum depth	9
	Minimum samples split	2 for cohort 1; 3 for the rest
	Maximum features	14
**Support vector machine**		
	C^b^	50
	Gamma	Auto, scale
	Decision function shape	One versus rest
	Kernel	Radial
**Artificial neural network**		
	Solver	Adam
	Learning rate	Adaptive
	Activation	Logistic
	Alpha	1e-2,1e-6
	Hidden layers	*4*: 70, 35, 30, 15; *5*: 60, 30, 30, 15, 10
**Adaptive boosting**		
	Base learner	RF
	Number of estimators	401
	Learning rate	1
	Algorithm	Samme.R
**Logistic regression**		
	Penalty	l2
	C	10
	Class weight	Balanced
	Max iteration	1000
	Solver	Sag

^a^RF: random forest.

^b^C: regularization parameter.

#### Random Forest

Random forest (RF) was used as both a standalone classifier and a base learner for the adaptive boosting (AdaBoost) algorithm. It has been widely used to predict survival data [26,27].

#### AdaBoost

The AdaBoost algorithm was applied to two weak learners, RF and LR. The study by Thongkam et al [34] used this algorithm on a breast cancer data set, where it outperformed all single classifiers. In our experiments, LR did not perform well; therefore, we did not pursue it. RF, with the optimized hyperparameters, was used to train the boosting classifier with a number of estimators and learning rates.

#### Artificial Neural Network

A backpropagation algorithm was used to train a neural network–based binary classifier. Generally, artificial neural networks (ANNs) perform well on survival data sets [30,35].

#### Support Vector Machines

Classification models using support vector machines (SVMs) have been applied to predict survival data [29,31,32]. To train the SVM, we experimented with different kernels, that is, linear, radial, sigmoid, and polynomial kernels. The linear and sigmoid kernels provided the lowest prediction scores; therefore, we did not use them further. A polynomial kernel with degree 2 yielded suboptimal results, and the SVM model could not converge for degree 3. The radial basis kernel was the most effective for learning the classification model.

### Calculating Feature Importance Over Time

The nonoverlapped time cohorts were used to calculate the feature importance scores to understand the changing relevance of features over time. We calculated these scores by training an RF classifier on the complete data set. The scores were calculated using Gini. Feature influence scores were used to understand the effect of features over the three cohorts.

## Results

### Overview

Below, we present the prediction performance of the four ML classifiers using both overlapped and nonoverlapped cohorts. As LR has been extensively used to predict time-to-event in organ transplant studies [16,29], it is used as a comparator classifier to the ML-based classifiers. The prediction performance of each of the best-trained classifiers (SVM, RF, AdaBoost, ANN, and LR), covering the three different time-to-event periods for both the original and reduced feature sets, were evaluated using 10-fold stratified cross validation for both overlapped and nonoverlapped cohorts. The results of each classifier were examined using the area under the curve (AUC) and F1 scores. The AUC score was used as the main performance evaluation metric to select the best model in each cohort and to make comparisons with similar studies. For our purpose, the ideal prediction model provides the best accuracy for graft failure. Therefore, to further substantiate the selection of the best model, we also evaluated the F1 score for graft failures. In cases where the AUC score was the same for different models, preference was given to the model with the highest F1 score.

### Analysis of Feature Engineering

Table 4 presents the results of feature engineering, whereby the prediction scores of all classifiers were obtained using both the original feature set and the reduced set. The reduced set consists of original continuous features and latent features returned by the stacked autoencoders. Cohort 1 for overlapped and nonoverlapped cohorts was the same; hence, the results were presented only once to avoid unnecessary duplication.

**Table 4:** Area under the curve comparison—all features with auto-encoded features.

Cohort			Overlapped				Nonoverlapped		
			All features (%)		Continuous+auto-encoded features (%)		All features (%)		Continuous+auto-encoded features (%)
**Cohort 1**									
	SVM^a^	80		82		N/A^b^		N/A	
	AdaBoost^c^	76		78		N/A		N/A	
	RF^d^	68		70		N/A		N/A	
	ANN^e^	62		61		N/A		N/A	
	LR^f^	62		62		N/A		N/A	
**Cohort 2**									
	SVM	63		66		53		53	
	AdaBoost	67		69		64		60	
	RF	62		65		65		67	
	ANN	62		62		62		62	
	LR	62		62		64		61	
**Cohort 3**									
	SVM	73		80		68		65	
	AdaBoost	76		81		68		64	
	RF	72		75		68		66	
	ANN	73		72		68		65	
	LR	69		69		62		64	

^a^SVM: support vector machine.

^b^N/A: not applicable.

^c^AdaBoost: adaptive boosting.

^d^RF: random forest.

^e^ANN: artificial neural network.

^f^LR: logistic regression.

The AUC scores (Table 4) show that prediction models for overlapped cohorts trained with auto-encoded features improved the prediction performance as compared with the prediction models trained using the original feature set. However, it was the opposite for nonoverlapped cohorts. Interestingly, the traditional approach of overlapped cohorts performed better with both the original and reduced feature sets compared with the nonoverlapped cohorts. Except for RF in cohort 2 of nonoverlapped cohorts, which showed slightly better performance (67%) when compared with its overlapped counterpart (65%), all other prediction models had better AUC scores with the traditional overlapping cohort approach. Therefore, for further analysis, we proceeded with overlapping cohorts only.

Although ANN and LR (the baseline model) showed no significant improvement across all three cohorts, the results confirmed the effectiveness of our deep learning architecture of stacked autoencoders for feature selection. For the subsequent prediction modeling analysis, we used the reduced feature set.

### Analysis of Prediction Performance of ML Models

Table 5 presents the prediction performance of the classifiers for each cohort in terms of AUC, F1 scores, recall, and precision, with SD for the 10-fold classification.

**Table 5:** Prediction performance of the machine learning classifiers across three different temporal cohorts using the overlapped patient stratification^a^.

Cohort			Auto-encoded feature set						
			AUC^b^ (%), mean (SD)		F1 (%), mean (SD)		Recall (%), mean (SD)		Precision (%), mean (SD)
**Cohort 1**									
	*SVM* ^c^	*82 (0.01)*		*61 (0.01)*		*49 (0.01)*		*90 (0.01)*	
	AdaBoost^d^	78 (0.01)		56 (0.01)		95 (0.01)		35 (0.01)	
	RF^e^	70 (0.009)		45 (0.001)		47 (0.01)		41 (0.01)	
	ANN^f^	61 (0.01)		5 (0.001)		42 (0.01)		6 (0.004)	
	LR^g^	62 (0.008)		39 (0.009)		58 (0.01)		29 (0.04)	
**Cohort 2**									
	SVM	66 (0.006)		53 (0.01)		55 (0.01)		60 (0.01)	
	*AdaBoost*	*69 (0.01)*		*63 (0.01)*		*64 (0.003)*		*63 (0.004)*	
	RF	65 (0.009)		62 (0.01)		62 (0.01)		61 (0.01)	
	ANN	63 (0.007)		60 (0.04)		55 (0.09)		60 (0.01)	
	LR	62 (0.008)		59 (0.009)		58 (0.01)		60 (0.004)	
**Cohort 3**									
	SVM	80 (0.005)		83 (0.003)		76 (0.003)		96 (0.003)	
	*AdaBoost*	*81 (0.01)*		*81 (0.004)*		*76 (0.003)*		*86 (0.01)*	
	RF	75 (0.008)		75 (0.006)		75 (0.01)		73 (0.01)	
	ANN	72 (0.007)		68 (0.005)		81 (0.03)		69 (0.01)	
	LR	69 (0.001)		77 (0.009)		70 (0.01)		70 (0.001)	

^a^Italics show the classifiers with the highest performance among the three cohorts.

^b^AUC: area under the curve.

^c^SVM: support vector machine.

^d^AdaBoost: adaptive boosting.

^e^RF: random forest.

^f^ANN: artificial neural network.

^g^LR: logistic regression.

**Figure 4:**
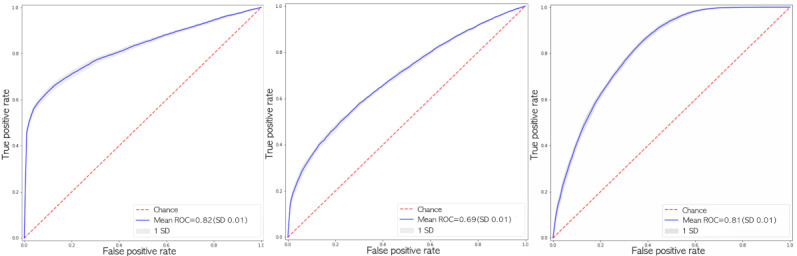
Receiver operating characteristic curves for support vector machine, adaptive boosting, and adaptive boosting for the three cohorts, respectively (left to right). AUC: area under the curve; ROC: receiver operating characteristic.

The classifiers performed differently across the three cohorts—SVM offered the highest prediction performance for short-term predictions, that is, for cohort 1, whereas AdaBoost offered the highest performance for the remaining cohorts. The SD across the different folds was nominal, confirming the stability of the classifiers. Figure 4 shows the receiver operating characteristic curves for the best models from each cohort.

To further investigate the prediction efficacy of the ML-based classifiers, we evaluated the prediction performance of the best-performing classifier for all three cohorts by testing the prediction of graft failure events by a classifier trained for a specific cohort with data from other cohorts, that is, testing the classifier for cohort 2 with randomly selected data from cohorts 1 and 3. The underlying assumption is that the classifier should not produce good prediction results for data from other cohorts. As this evaluation considers survivors across progressive cohorts, we used the F1 score to measure prediction performance. A sound prediction model for cohort 2 will give a high graft failure prediction score for data from cohort 1 but a low prediction score for data from cohort 3, the rationale being that the overlapping cohort 2 classifier is trained on graft failure cases in both cohorts 1 and 2*.* Therefore, the prediction model for cohort 2 should give a high prediction score for predicting graft failures from year_0_ to year_5_, but when applied to cohort 3*,* the cohort 2 prediction model would be unable to predict graft failure as it has not been trained on cohort 3 data. Table 6 provides the cross-cohort prediction scores for the best classifiers for each cohort.

**Table 6:** Prediction performance (F1 scores) for cross-cohort predictions using overlapped cohorts.

Model	Cohort 1	Cohort 2	Cohort 3
SVM^a^ (cohort 1)	0.6	0.42	0.29
AdaBoost^b^ (cohort 2)	0.79	0.87	0.58
AdaBoost (cohort 3)	0.72	0.75	0.87

^a^SVM: support vector machine.

^b^AdaBoost: adaptive boosting.

### Results of Wilcoxon Signed-Rank Test

To determine if the prediction differences between the different models were statistically significant, we used the Wilcoxon signed-rank test to compare the scores between different models. Because the best scores in each cohort were usually produced by SVM and AdaBoost models, the Wilcoxon signed-rank test was conducted with each combination of these models with the other models.

Table 7 shows the results based on the F1 score, and the *P* values between the models were quite small and less than the threshold value of *P*=.05, confirming that the performance difference is statistically significant.

**Table 7:** The results for Wilcoxon signed-rank test (F1).

Cohort	*P* value (F1)				
	SVM^a^-AdaBoost^b,c^	SVM-ANN^d,e^	SVM-RF^f,g^	AdaBoost-RF^h^	AdaBoost-ANN^i^
Cohort 1	.003	.003	.003	.003	.003
Cohort 2	.003	.003	.003	.003	.03
Cohort 3	<.001	<.001	<.001	<.001	<.001

^a^SVM: support vector machine.

^b^AdaBoost: adaptive boosting.

^c^H_o_ (null hypothesis): SVM=AdaBoost; H_a_ (alternative hypothesis): SVM≠AdaBoost.

^d^ANN: artificial neural network.

^e^H_o_: SVM=EANN; H_a_: SVM≠EANN.

^f^RF: random forest.

^g^H_o_: SVM=RF; H_a_: SVM≠RF.

^h^H_o_: AdaBoost=RF; H_a_: AdaBoost≠RF.

^i^H_o_: AdaBoost=ANN; H_a_: AdaBoost≠ANN.

### Analysis of the Influence of Clinical Features Over Time Toward Graft Status Prediction

#### Overview

The second objective of this research is to analyze the influence of clinical features on the prediction of graft survival over different periods. The intent was to understand the factors responsible for graft survival at different periods after transplant. The nonoverlapped cohorts (0-1 years, >1-5 years, and >5-17 years following a transplant) were used to ensure that there was no cascading influence of the features over time. For comparison purposes, we also examined the feature importance for overlapping cohorts. The feature importance scores represent the relative importance of the feature among all features, that is, the total of all the features’ importance scores add up to 100%; hence, if one feature gains a higher importance score, it will be at the expense of the importance score of other features.

**Figure 5:**
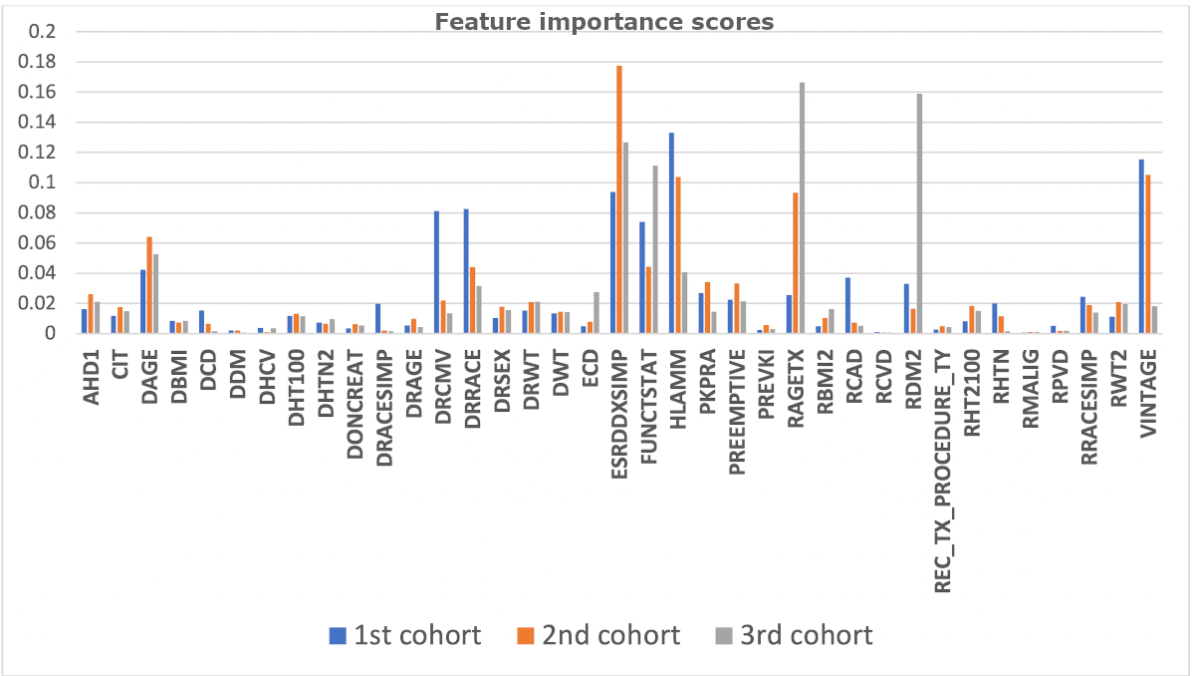
Changing relevance of features based on nonoverlapped time cohorts.

**Figure 6:**
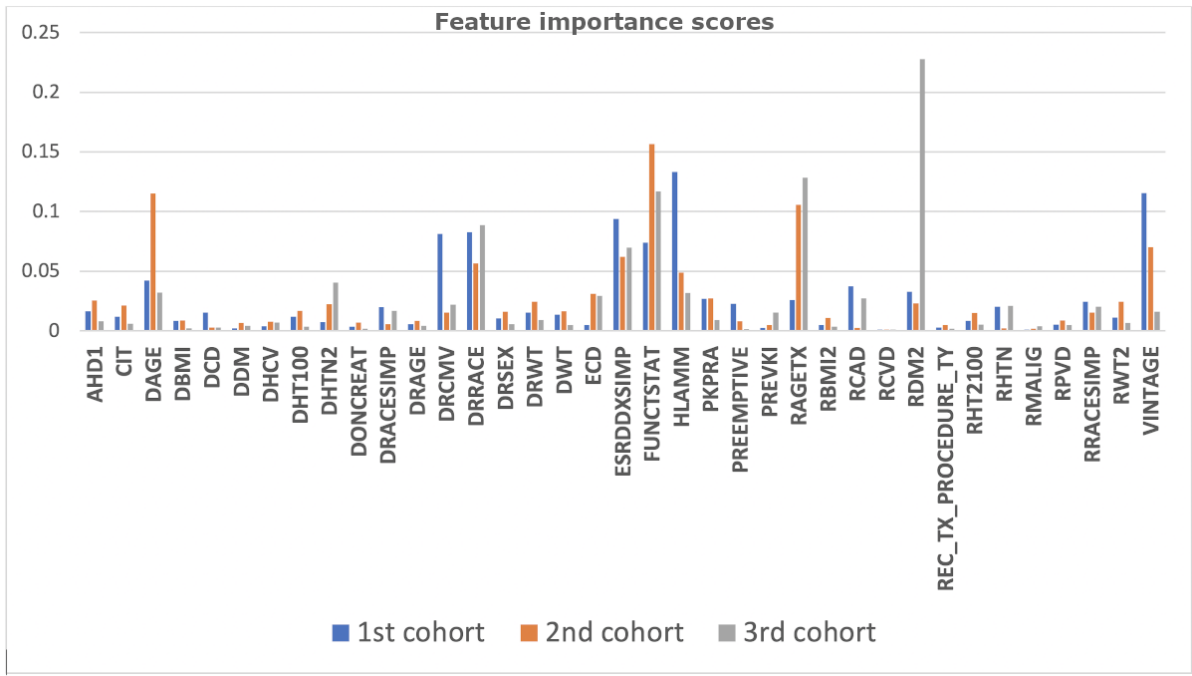
Changing relevance of features based on overlapped time cohorts.

Figures 5 and 6 illustrate the individual feature importance scores across all the nonoverlapped and overlapped cohorts, respectively.

In general, the top 10% of the important features remained consistent in both the nonoverlapped and overlapped cohorts; however, we note that the nonoverlapped cohorts identified a larger group of important features. For instance, peak panel reactive antibody (Pkpra) and pre-emptive recipient status (Preemptive) had negligible importance in overlapping cohorts but were important during the 2-5 years and 6-17 years in the nonoverlapping cohorts. Table 8 shows the feature importance across the three cohorts.

**Table 8:** Ranking of the top-10 features across the time cohorts with feature importance scores^a^.

Rank	Cohort 1, feature, relative score (%)	Cohort 2		Cohort 3	
		Feature, relative score (%)	Importance (%), rank change	Feature, relative score (%)	Importance (%), rank change
1	HLAMM^b^ (13)	ESRDDXSIMP^c^ (18)	*+100, +2*	RAGETX^d^ (16)	+77, *+3*
2	VINTAGE^e^ (12)	VINTAGE (11)	–8, *0*	RDM2^f^ (16)	>+100, *>+10*
3	ESRDDXSIMP (9)	HLAMM (10)	–23, –*2*	ESRDDXSIMP (13)	–27, –*2*
4	DRCMV^g^ (8)	RAGETX (9)	+78, *+6*	FUNCTSTAT^h^ (12)	>+100, *+3*
5	DRRACE^i^ (8)	DAGE^j^ (6)	50, *+2*	DAGE (5)	–16, *0*
6	FUNCTSTAT (7)	DRRACE (4)	–100, –*1*	DRRACE (3)	–25, *0*
7	DAGE (4)	FUNCTSTAT (4)	–75, –*1*	ECD^k^ (3)	>+100, *>+3*
8	RCAD^l^ (4)	PKPRA^m^ (3)	+50, *>+3*	VINTAGE (2)	>–100, –*6*
9	RDM2 (3)	PREEMPTIVE^n^ (3)	+50, *>+1*	PREEMPTIVE (2)	–33, *0*
10	RAGETX (2)	DRCMV (2)	–75, –*6*	RWT2 (2)	0, *0*
Rest	Rest (31)	Rest (30)	Rest (30)	Rest (26)	Rest (26)

^a^Importance (%) and rank change is shown in italics.

^b^HLAMM: HLA antigen mismatch.

^c^ESRDDXSIMP: simplified end-stage renal disease diagnosis.

^d^RAGETX: recipient age.

^e^VINTAGE: number of years on dialysis before transplant.

^f^RDM2: recipient diabetes status.

^g^DRCMV: donor-recipient cytomegalovirus.

^h^FUNCTSTAT: functional status of the recipient.

^i^DRRACE: donor-recipient race.

^j^DAGE: donor age.

^k^ECD: expanded criteria donor.

^l^RCAD: recipient coronary artery disease.

^m^PKPRA: peak panel reactive antibody.

^n^PREEMPTIVE: pre-emptive transplant.

^o^RWT2: recipient weight.

Below, we analyze the importance of features in each cohort and show the influence of features over time using nonoverlapping cohorts.

#### Feature Importance for Cohort 1

According to the top features shown in Table 8, HLAMMs and the number of years on dialysis before transplant (VINTAGE) were the most important features with a relative importance of over 10%. This observation has been confirmed in other studies [32,36]. Donor-recipient CMV status, donor-recipient race, end-stage renal disease diagnosis (ESRDDXSIMP), and functional status of the recipient were ranked as having medium importance with a relative score between 5% and 10%. Donor-recipient race pairs and donor-recipient CMV pairs were noted to have more predictive influence in cohort 1 than in the other two cohorts.

#### Feature Importance for Cohort 2

Both HLAMMs and VINTAGE remained highly important in cohort 2. In addition, ESRDDXSIMP was noted as a highly important feature. Interestingly, we note that few features, such as donor age and recipient age, were rather insignificant in cohort 1 but were noted to be significant in both cohort 2 and further in cohort 3.

#### Feature Importance for Cohort 3

ESRDDXSIMP showed a relative downward trend; however, it remained a highly significant feature. Unlike earlier cohorts, HLAMMs and VINTAGE were noted to not maintain their importance in the long term, whereas the recipient’s status of diabetes was noted to be the most important feature, along with recipient age and their functional status. Donor age was noted to maintain a medium importance score between 5% and 10%.

**Figure 7:**
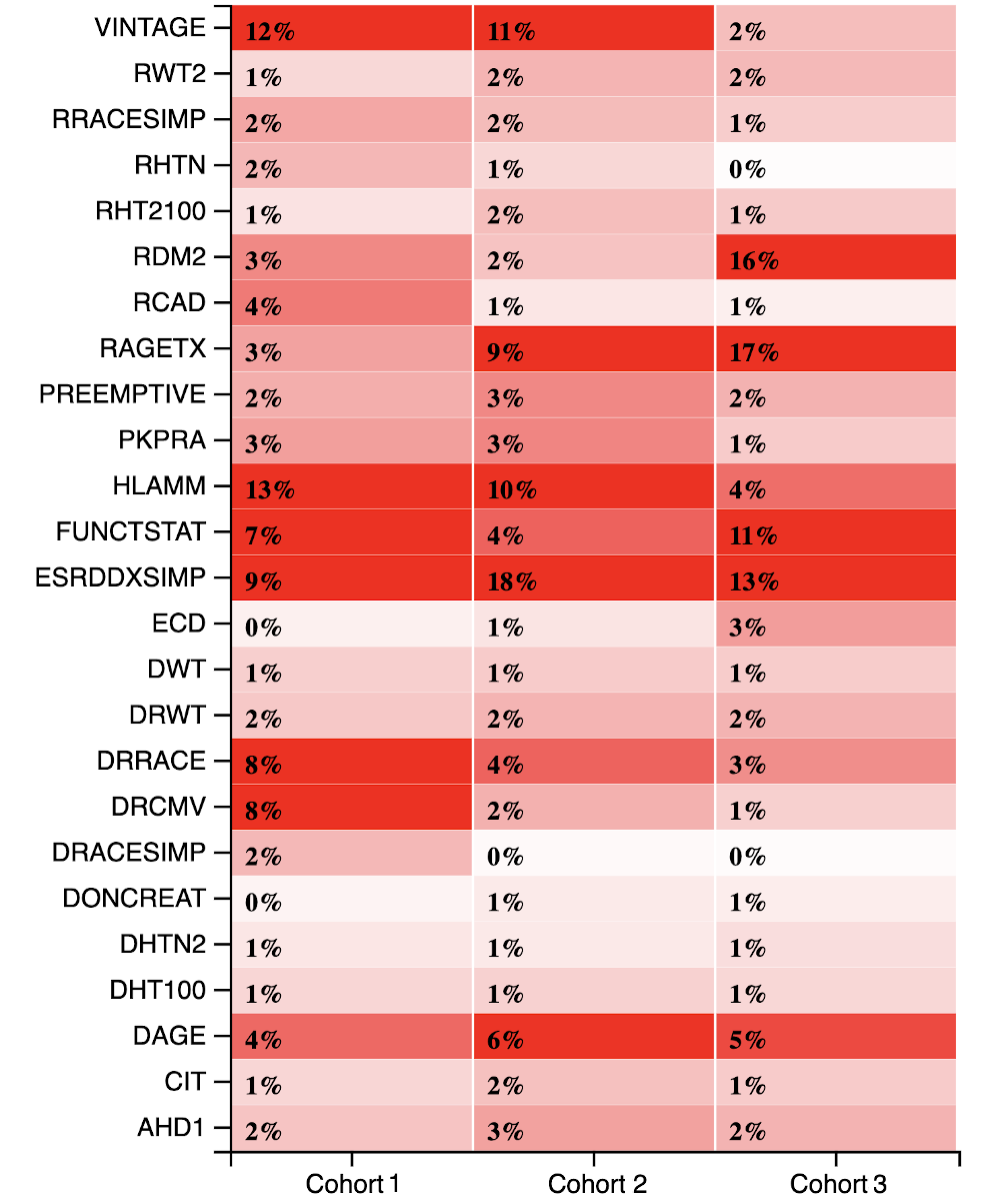
Changing relevance of top 25 features over the three cohorts. AHD1: donor-recipient height difference; CIT: cold ischemia time; DAGE: donor age; DHT100: donor height; DHTN2: donor hypertension; DONCREAT: donor creatinine level; DRACESIMP: donor race; DRCMV: donor-recipient cytomegalovirus; DRRACE: donor-recipient race; DRWT: donor-recipient weight difference; DWT: donor weight; ECD: expanded criteria donor; ESRDDXSIMP: simplified end-stage renal disease diagnosis; FUNCTSTAT: functional status of the recipient; HLAMM: number of HLA mismatches; PKPRA: peak panel reactive antibody; PREEMPTIVE: preemptive transplant; RAGETX: recipient age; RCAD: recipient coronary artery disease; RDM2: recipient diabetes; RHT2100: recipient height; RHTN: recipient hypertension; RRACESIMP: recipient race; RWT2: recipient weight; VINTAGE: number of years on dialysis before transplantation.

Figure 7 presents a heat map of the importance score to illustrate the changing influence of the top 25 features across the three cohorts. In addition to the top 10 features (Table 8), the heat map details the contribution of relatively less important features. It was interesting to see that few features (such as donor weight, recipient weight, and donor hypertension) had static importance across the three cohorts. This indicates that although these features were not deterministic for the cohorts, they possessed a certain value for the prediction models.

**Figure 8:**
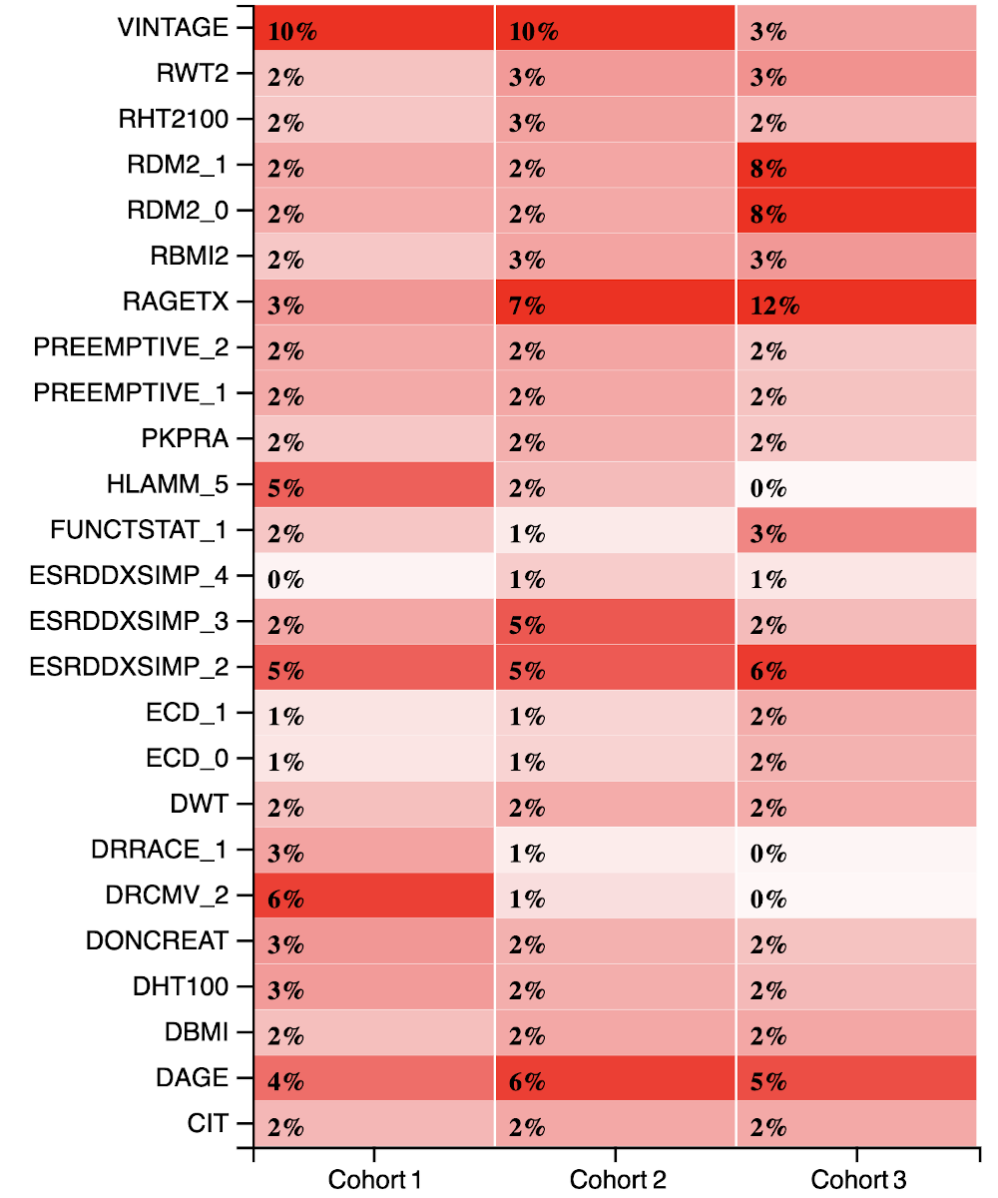
Changing relevance of top 25 features (including dummy features) over the three cohorts. CIT: cold ischemia time; DAGE: donor age; DBMI: donor BMI; DHT100: donor height; DONCREAT: donor creatinine level; DRCMV_2: Donor positive recipient positive; DRRACE_1: Donor white recipient white; DWT: donor weight; ECD_0: Expanded criteria donor: no; ECD_1: Expanded criteria donor: yes; ESRDDXSIMP_2: End stage renal disease: diabetes mellitus; ESRDDXSIMP_3: End stage renal disease: polycystic kidney disease; ESRDDXSIMP_4: End stage renal disease: hypertension; FUNCTSTAT_1: Functional status of recipient: 100% no complaints; HLAMM _5: Number of human leukocyte antigen mismatches: 5; PKPRA: peak panel reactive antibody; PREEMPTIVE_1: Preemptive transplant: yes; PREEMPTIVE_2: Preemptive transplant: no; RAGETX: recipient age; RBMI2: recipient BMI; RDM2_0: Recipient diabetes: no; RDM2_1: Recipient diabetes: yes; RHT2100: recipient height; RWT2: recipient weight; VINTAGE: number of years on dialysis before transplantation.

Analysis of the values of categorical features provided novel insights into the influence of a feature. Figure 8 presents a heat map of the importance of the value of the categorical features generated after transforming them into dummy features.

## Discussion

### Principal Findings

The cross-cohort prediction results (Table 6) confirm the efficacy of the classifiers—the prediction model for cohort 3 (ie, AdaBoost) correctly offers a high prediction score for data from cohort 1 (72%) and cohort 2 (75%). The prediction model for cohort 2 offers a high prediction score for cohort 1 data (79%) but a low prediction score for cohort 3 (58%) data. The classifier for cohort 1 (ie, SVM) gave low prediction scores for data from cohort 2 (42%) and cohort 3 (29%). Interestingly, the highest prediction score by a cohort-specific classifier was always achieved for data from its respective cohort. The prediction modeling results confirmed that the prediction models were highly sensitive to their respective cohorts.

### Comparing Prediction Performance With Prior Studies

We compared the prediction performance of our ML-based prediction models with comparable organ transplant studies that involved similar-sized observations and temporal windows. Table 9 summarizes the findings of the two studies for each cohort. There have been several other studies [19,31,32,35,37,38] to predict the short-term graft status of different organ transplants, but because of their small data set, these do not serve as a meaningful comparison.

**Table 9:** Prediction scores of similar studies.

Time	Study	Model	Size	Data set	Metric	Score (%)	Our score (%)
1 year	Lin et al [16]	ANN^a^ and LR^b^	46,414	UNOS^c^	AUC^d^	73	82
1 year	Dag et al [23]	LR	15,580	UNOS	AUC	63	82
5 years	Tiong et al [39]	Nomogram	20,085	UNOS	C-index^e^	71	69
5 years	Lin et al [16]	ANN	17,856	UNOS	AUC	77	69
7 years	Lin et al [16]	ANN	10,250	UNOS	AUC	82	81
14 years	Luck et al [40]	ANN	46,098	SRTR^f^	C-index	65	81

^a^ANN: artificial neural network.

^b^LR: logistic regression.

^c^UNOS: United Network of Organ Sharing.

^d^AUC: area under the curve.

^e^C-index: concordance index.

^f^SRTR: Scientific Registry of Transplant Recipients.

When comparing our results with prior studies, it is noted that although our cohort 2 prediction performance (ie, graft status prediction over a 5-year period) is lower than that of Lin et al [16], it was based on a much smaller data set that included 10,641 survivals and 7215 failures, whereas we analyzed 23,475 failures and 29,352 survivals. Similarly, Tiong et al [39] analyzed a smaller sample of 20,085 living donor transplant recipients to achieve a concordance index of 71%. Our cohort 3 prediction performance is marginally lower compared with Lin et al [16], who predicted a 7-year graft survival with an 82% AUC score, whereas our cohort 3 prediction model covers a much longer (17 years) temporal window and achieves a comparable prediction score. Using a similar number of transplants, Luck et al [40] achieved a much lower concordance index between 63% and 66% for 14-years graft survival.

### Limitations and Future Work

A limitation of our research lies in the removal of censored instances. We removed all successful cases that were censored before 8 years following transplant. Although this type of approach has previously been used, including censored cases is a potential consideration for future analyses.

### Conclusions

Understanding the impact of donor and recipient factors that predict short- and long-term kidney transplant allograft survival is important for patients and providers. Kidney transplantation is the optimal form of kidney replacement therapy, but kidney allografts are a limited resource. In addition, the alternative to kidney transplantation (ie, dialysis) is considerably costlier.

In this study, we present an ML-based framework to predict the status of kidney allografts, based on donor-recipient features, over a period of 17 years. We applied ML-based data analysis methods for feature engineering to reduce data dimensionality, develop prediction models for three distinct temporal cohorts, and investigate the changing relevance of clinical features across different temporal cohorts. We introduced the concept of nonoverlapped cohorts to analyze the changing relevance of features in three defined periods. In conclusion, our results emphasize that ML can be effective in predicting graft survival using donor and recipient factors that are routinely collected as part of patient care. As a next step, we plan to incorporate the prediction models into clinical care at the time of allocation; models that best predict short- and long-term kidney graft survival may be used as a pragmatic prognostic tool to aid clinicians in maximizing the best possible matching of donors and recipients while preserving existing allocation rules that are used to promote equity [41].

## Appendix

Multimedia Appendix 1 Supplementary tables.

## Abbreviations

AdaBoost: adaptive boosting
ANN: artificial neural network
AUC: area under the curve
CMV: cytomegalovirus
ESRDDXSIMP: end-stage renal disease diagnosis
LR: logistic regression
ML: machine learning
RF: random forest
SMOTE: Synthetic Minority Oversampling Technique
SRTR: Scientific Registry of Transplant Recipients
SVM: support vector machine
VINTAGE: number of years on dialysis before transplant
