# Supplementary material for: Predicting Kidney Graft Survival Using Machine Learning Methods: Prediction Model Development and Feature Significance Analysis Study
Source: J Med Internet Res. 2021 Aug 27;23(8):e26843. doi: 10.2196/26843 (PMC8433864; doi:10.2196/26843)
Supplement: Multimedia Appendix 1 [file jmir_v23i8e26843_app1.doc]

# Multimedia Appendix 1: Supplementary Tables

**Table S1.** Results with Stacked Autoencoders using Continuous features as input

| Results with Auto-Encoded Continuous Features Only | | | | |
| --- | --- | --- | --- | --- |
|  | AUC | F1 | Recall | Precision |
|  | | | | |
| **Cohort 1** | | | | |
| SVM | 71% | 47% | 37% | 62% |
| AdaBoost | 70% | 42% | 58% | 33% |
| RF | 70% | 46% | 38% | 57% |
| ANN | 35% | 0 | 0 | 0 |
| LR | 68% | 44% | 33% | 65% |
| **Cohort 2** | | | | |
| SVM | 65% | 61% | 60% | 62% |
| AdaBoost | 62% | 59% | 59% | 58% |
| RF | 65% | 60% | 60% | 61% |
| ANN |  |  |  |  |
| LR | 64% | 61% | 60% | 62% |
| **Cohort 3** | | | | |
| SVM | 76% | 73% | 76% | 70% |
| AdaBoost | 75% | 76% | 73% | 79% |
| RF | 76% | 72% | 75% | 70% |
| ANN | 74% | 73% | 74% | 73% |
| LR | 75% | 70% | 76% | 65% |

**Table S2**. Hyperparameters tested during random search for optimal combinations.

| Method | Hyper-Parameter | Random Values |
| --- | --- | --- |
|  |  |  |
| Random Forest | Number of estimators | 25,50,100,200,250 |
|  | Class weight | Balanced |
|  | Criterion | Gini, Entropy |
|  | Max Depth | 5,9,14 |
|  | Min samples split | 2 for cohort1,3 for rest |
|  | Max features | 5,9,14 |
| Support Vector Machine | C | 1,10,50,100 |
|  | Gamma | Auto, scale |
|  | Decision function shape | One vs rest |
|  | Kernel | Radial, Sigmoid, Polynomial |
| Artificial Neural Network | Solver | Adam |
|  | Learning rate | Adaptive |
|  | Activation | Logistic, Relu |
|  | Alpha | 1e-2,1e-6 |
|  | Hidden layers | **4**:70,35,30,15  **5**: 60,30,30,15,10 |
| Adaptive Boosting | Base learner | RF |
|  | Number of estimators | 350, 375, 401, 450 |
|  | Learning rate | 1 |
|  | Algorithm | Samme.R |
| Logistic Regression | Penalty | l2 |
|  | C | 10 |
|  | Class weight | Balanced |
|  | Max iteration | 1000 |
|  | Solver | Sag |

**Table S3**. Results with imbalanced dataset

| Results for Overlapped Cohorts Without Oversampling | | | | |
| --- | --- | --- | --- | --- |
|  | AUC | F1 | Recall | Precision |
|  | | | | |
| **Cohort 1** | | | | |
| SVM | 62% | 29% | 53% | 20% |
| AdaBoost | 55% | 0.2% | 0.5% | 0.1% |
| RF | 60% | 24% | 26% | 21% |
| ANN | 61% | 0 | 0 | 0 |
| LR | 61% | 29% | 59% | 19% |
| **Cohort 2** | | | | |
| SVM | 66% | 53% | 55% | 60% |
| AdaBoost | 69% | 63% | 64% | 63% |
| RF | 65% | 62% | 62% | 61% |
| ANN | 63% | 60% | 55% | 60% |
| LR | 62% | 59% | 58% | 60% |
| **Cohort 3** | | | | |
| SVM | 57% | 83% | 72% | 97% |
| AdaBoost | 66% | 82% | 73% | 93% |
| RF | 68% | 75% | 79% | 71% |
| ANN | 67% | 83% | 73% | 95% |
| LR | 66% | 71% | 80% | 63% |

**Table S4**. Feature description and abbreviations for figure 8

| Feature description | Abbreviation |
| --- | --- |
| Years on dialysis pretransplant | VINTAGE |
| Recipient weight | RWT2 |
| Recipient height | RHT2100 |
| Donor age | DAGE |
| Donor height | DHT100 |
| Donor weight | DWT |
| Donor creatinine level | DONCREAT |
| Expanded criteria donor: yes | ECD_1 |
| Expanded criteria donor: no | ECD_0 |
| Recipient body mass index | RBMI2 |
| Donor body mass index | DBMI |
| Cold ischemia time | CIT |
| Recipient age | RAGETX |
| Number of human leukocyte antigen mismatches: 5 | HLAMM_5 |
| Functional status of recipient: 100% no complaints | FUNCTSTAT_1 |
| Donor positive recipient positive | DRCMV_2 |
| Donor white recipient white | DRRACE_1 |
| Peak panel reactive antibody | PKPRA |
| End stage renal disease: hypertension | ESRDDXSIMP_4 |
| End stage renal disease: polycystic kidney disease | ESRDDXSIMP_3 |
| End stage renal disease: diabetes mellitus | ESRDDXSIMP_2 |
| Preemptive transplant: no | PREEMPTIVE_2 |
| Preemptive transplant: yes | PREEMPTIVE_1 |
| Recipient diabetes: yes | RDM2_1 |
| Recipient diabetes: no | RDM2_0 |
